# Association of maxillofacial injuries with traumatic brain injuries in paediatric patients: a case–control study

**DOI:** 10.1186/s12903-024-05366-4

**Published:** 2024-12-26

**Authors:** Baranya Shrikrishna Suprabha, Michael Lowery Wilson, Joanna Baptist, Sadhvi Shankar Subramanian, Ramya Shenoy, Fatemeh Jahanjoo, Jeedhu Radhakrishnan, V. Mayur Kamath, N. P. Anagha, Diksha Chaurasia

**Affiliations:** 1 Department of Pediatric and Preventive Dentistry, Manipal College of Dental Sciences Mangalore, Manipal Academy of Higher Education, Manipal, Karnataka 576104 India; 2 Injury Epidemiology and Prevention Research Group, Turku Brain Injury Center, Division of Clinical Neurosciences, Turku University Hospital and University of Turku, Turku, Finland; 3 Section for Oral Health, Heidelberg Institute of Global Health, University of Heidelberg, Im Neuenheimer Feld 130.3, 69120 Heidelberg, Germany; 4 Department of Oral and Maxillofacial Surgery, Manipal College of Dental Sciences Mangalore, Manipal Academy of Higher Education, Manipal, Karnataka 576104 India; 5 Turku University Hospital and University of Turku, Turku, Finland; 6 Department of Public Health Dentistry, Manipal College of Dental Sciences Mangalore, Manipal Academy of Higher Education, Manipal, Karnataka 576104 India; 7 Physical Medicine and Rehabilitation Research Centre, Aging Research Institute, Tabriz University of Medical Sciences, Tabriz, Iran; 8 Department of Emergency Medicine, Kasturba Medical College, Mangalore, Manipal Academy of Higher Education, Manipal, Karnataka 576104 India; 9 Department of Neurosurgery, Kasturba Medical College, Mangalore, Manipal Academy of Higher Education, Manipal, Karnataka 576104 India; 10 Manipal College of Dental Sciences Mangalore, Manipal Academy of Higher Education, Manipal, Karnataka 576104 India

**Keywords:** Traumatic brain injury, Maxillofacial trauma, Paediatric, Children

## Abstract

**Background:**

Traumatic brain injuries (TBIs) are among the most challenging conditions to accurately diagnose in children, and many TBIs are underdiagnosed. Patients with maxillofacial injury may be at risk for TBI. The objective of this study was to analyse the association between maxillofacial injuries and TBI among paediatric patients. Analysis of the factors associated with the occurrence of TBI among paediatric patients with maxillofacial injuries was the secondary objective.

**Method:**

This case-control study included 192 cases defined as paediatric patients with maxillofacial injuries and 192 controls defined as all paediatric patients with traumatic injuries to other body locations and with no maxillofacial injuries. The medical records of patients aged 0–18 years, who underwent treatment at a tertiary care hospital were screened. Patient demographic data, causes of trauma, and the presence, type, and severity of TBI were recorded. For the cases, the presence of facial fracture, type and severity of the maxillofacial injuries were recorded. The odds ratio was obtained to determine the association between maxillofacial injuries and TBI. Multiple logistic regression analysis was performed to study the associations of all the recorded study variables with the occurrence of TBI in patients with maxillofacial injuries.

**Results:**

A statistically significant difference was seen between the cases and controls for TBI occurrence, with 53% of cases recording TBI, compared to 32% among controls, with an odds ratio of 2.5. Multiple logistic regression analysis with the presence/absence of TBI among the cases as the dependent variable revealed a significant association of road traffic accidents (RTA), longer duration of hospital stays, and dentoalveolar fracture with TBI.

**Conclusion:**

Paediatric patients with maxillofacial injuries need to be screened for TBI, as the risk of occurrence of TBI is 2.5 times greater in these patients than in those with injuries involving other parts of the body. RTA and prolonged hospitalization are factors associated with TBI among paediatric patients with maxillofacial injuries. The presence of only dentoalveolar injuries decreases the likelihood of a TBI.

## Background

Traumatic brain injury (TBI) occurs when an external mechanical force distorts the normal function or structure of the brain [1]. TBIs are recognized as the leading cause of acquired disability and death for those under 18 years of age, with 865 per 100,000 children being diagnosed with a TBI annually [2, 3]. Over 500,000 children and adolescents under the age of 14 years sustain a TBI necessitating emergency department visit resulting in health care cost exceeding 1 billion USD per year [4].

A large proportion of TBIs diagnosed among paediatric populations are the result of falls, road traffic accidents and abuse [5]. Infants are particularly prone to falls that result in head trauma because of their larger head-to-body ratios, unsteady gait, and unbalanced ambulation [6].The physical abuse of children is also well represented as a risk factor for TBI among this population. Once children reach puberty, their risk profiles for TBI begin to shift toward patterns that reflect their increasing autonomy, risk-taking behaviour and greater distance from supervision. Injuries related to sports, substance use, bike collisions and attempted suicides are among the most common contributors to TBI among adolescents [7].

TBIs are among the most challenging conditions to accurately diagnose in children, and many TBIs are underdiagnosed. One of the potential areas of diagnostic focus could lie in paediatric patients who report to emergency centres with varying forms of facial trauma. There is some evidence that such patients may experience brain injury [8]. However, since not all symptoms of TBI are clinically obvious at the time the patient reports facial trauma, the potential exists for a missed diagnosis. Such missed cases of TBI may have long-term implications for the cognitive, behavioural and psychosocial development of the individual, and it is thus imperative that informed inquiries be made as to whether such cases are being missed [5].

A pilot study conducted in Malaysia revealed that nearly 40% of 11,294 patients admitted for maxillofacial trauma experienced traumatic brain injury [8]. A systematic review regarding the association of TBI with maxillofacial fractures showed that the global incidence of maxillofacial fractures with TBI ranged from 7.6% to approximately 87% of all maxillofacial injuries, with wide variation among studies conducted in different countries [9]. Few studies involving Indian populations have also reported a high incidence of TBI among patients reporting maxillofacial injuries [10–12]. While one research reported that most patients with TBI had fractures of the upper or the middle third of the face [11], another study reported that brain injuries are commonly associated with mid-face injuries [12]. These studies mostly involved adults, and there is a dearth of studies involving the paediatric population in India. The risk and manifestations of TBI differ among children due to age-related structural differences and injury mechanisms due to differences in physical ability based on age and problems associated with neurological evaluation [13].

Hence, we conducted a study with the objective of analysing the association between maxillofacial injuries and traumatic brain injury among paediatric patients in an Indian population. Analysis of the factors associated with the occurrence of TBI among patients with maxillofacial injuries was the secondary objective.

## Methods

This retrospective case–control study was conducted at a tertiary care hospital in Mangalore, Karnataka, which is in southern India. The study has been conducted and is reported in accordance with the STROBE guidelines for case-control studies.

### Ethical considerations

Ethics approval was obtained from the Institutional Ethics Committee of the Manipal College of Dental Sciences, Mangalore (Ref. no. 2044), after which the study commenced. Permission was obtained from the hospital authorities prior to the study. The patients’ identities were anonymized by assigning codes during data collection, and the confidentiality of the data was maintained throughout the study. The research was completed in accordance with the Declaration of Helsinki as revised in 2013.

#### Sampling

The medical records of patients reporting with trauma to Kasturba Medical College Hospital, Mangalore were screened to identify cases and controls and obtain relevant data on risk factors for TBI among patients with maxillofacial injuries.

#### Sample size calculation

To calculate the sample size, the proportion of exposure of the cases was assumed to be 43.88%, based on the data in the Indian population^9^, the least extreme odds ratio to be detected as 1.82 at a 95% confidence interval and 80% power, the ratio of case to control being 1:1. The sample size for cases was thus estimated to be 192, with an equal number of controls. The sample size was calculated using OpenEpi, Version 3, open-source calculator -SSCC (https://www.openepi.com/SampleSize/SSCC.htm).

#### Definition of cases

Paediatric patients reporting with maxillofacial injuries. Maxillofacial injury was defined as any injury involving hard tissue, soft tissue or both in the facial region extending from the frontal region to the mandibular region.

#### Definition of controls

All paediatric patients with traumatic injuries to other body locations and with no maxillofacial injuries.

#### Inclusion and exclusion criteria

The patient registers at the emergency department of the hospital were screened, and all patients aged 0–18 years reporting with traumatic injury were included in the study. The exclusion criteria were incomplete medical records [including computed tomography (CT) scan], TBI occurring in isolation and not associated with other traumatic injuries, and maxillofacial injuries along with injuries involving other body parts or organs (poly-trauma cases).

### Data collection

The patient register, and case records over a period of fifteen years (2008–2022) were screened to identify patients between 0 and 18 years of age reporting with traumatic injuries. Based on the injury record in the patient register, the samples were selected and narrowed down as per the definition of case and control. Patients were subsequently selected based on the exclusion criteria after reviewing the case records of the patients selected from the patient register. The demographic factors (age and sex), cause of trauma (road traffic accident (RTA), assault, sports-related injury, fall, assault/abuse), medical history or disability, drug/alcohol abuse history, symptoms related to TBI (vomiting, loss of consciousness, headache, amnesia, convulsions), the presence, type and severity of TBI were included in the data. Findings from neurological assessments, such as alterations or loss of consciousness, alterations in mental state (immediately related to the TBI), post-traumatic amnesia, Glassgow Coma Scale (GCS) scores (best score available in the first 24 h), and CT scan findings (normal/abnormal), were used to classify the severity of TBI. The severity of TBI was classified into 3 groups, mild, moderate, and severe, based on the criteria given by Rajandram et al.^8^ For mild TBI, the GCS score ranged from 13 to 15, and no abnormalities were detected on structural imaging, with a history of loss of consciousness for up to 30 min/alteration of mental state for up to 24 h/post traumatic amnesia for up to 1 day. A GCS score of 9–12 with abnormal structural imaging and a history of loss of consciousness > 30 min but < 24 h/abnormal mental state > 24 h/post traumatic amnesia > 1 day but < 7 days was classified as moderate TBI. A GCS score ≤ 8 with or without abnormal structural imaging and a history of loss of consciousness > 24 h/abnormal mental state > 24 h/post traumatic amnesia > 7 days was classified as severe TBI [8].

The records of paediatric patients with maxillofacial trauma treated at the hospital were further screened for data on the type and severity of maxillofacial injuries and hospitalization duration. The severity of maxillofacial injuries was scored based on the Facial Injury Severity Scale (FISS) [14]. Maxillofacial injuries were classified as injuries with and without facial fractures. In addition, the presence of soft tissue injuries, facial fractures and dentoalveolar injuries were noted. Facial fractures were divided into upper, midface and lower face types. Frontal bone fractures were included as upper face fractures, while maxillary, orbital, zygomatic, ethmoidal, and nasal bone fractures were categorized as midface fractures. Any fracture in the mandibular structure (symphysis, angle, body, condyle, parasymphysis) was included as a mandibular fracture [8]. All the data were recorded in a data sheet by two investigators.

### Data analysis

The data were analysed using the Statistical Package for Social Sciences (SPSS, Version 26, IBM, Chicago, IL, USA). To demonstrate the distribution of the trauma among various ages in the sample, the patients between 0 and 18 years of age were further subdivided into 3 categories namely 0–5 years (early childhood), 6–11 years (middle childhood) and 12–18 years (adolescence) at the data analysis stage. Descriptive data on percentage frequencies and mean FISS scores were obtained. The association between maxillofacial injuries and TBI was calculated using the chi-square test, and odds ratios were calculated. The frequencies related to demographic data, patient history, and type of injury were compared using the chi-square test. Multiple logistic regression analysis was performed to study the associations of all the recorded study variables with the occurrence of TBI in patients with maxillofacial injuries.

## Results

**Fig. 1:**
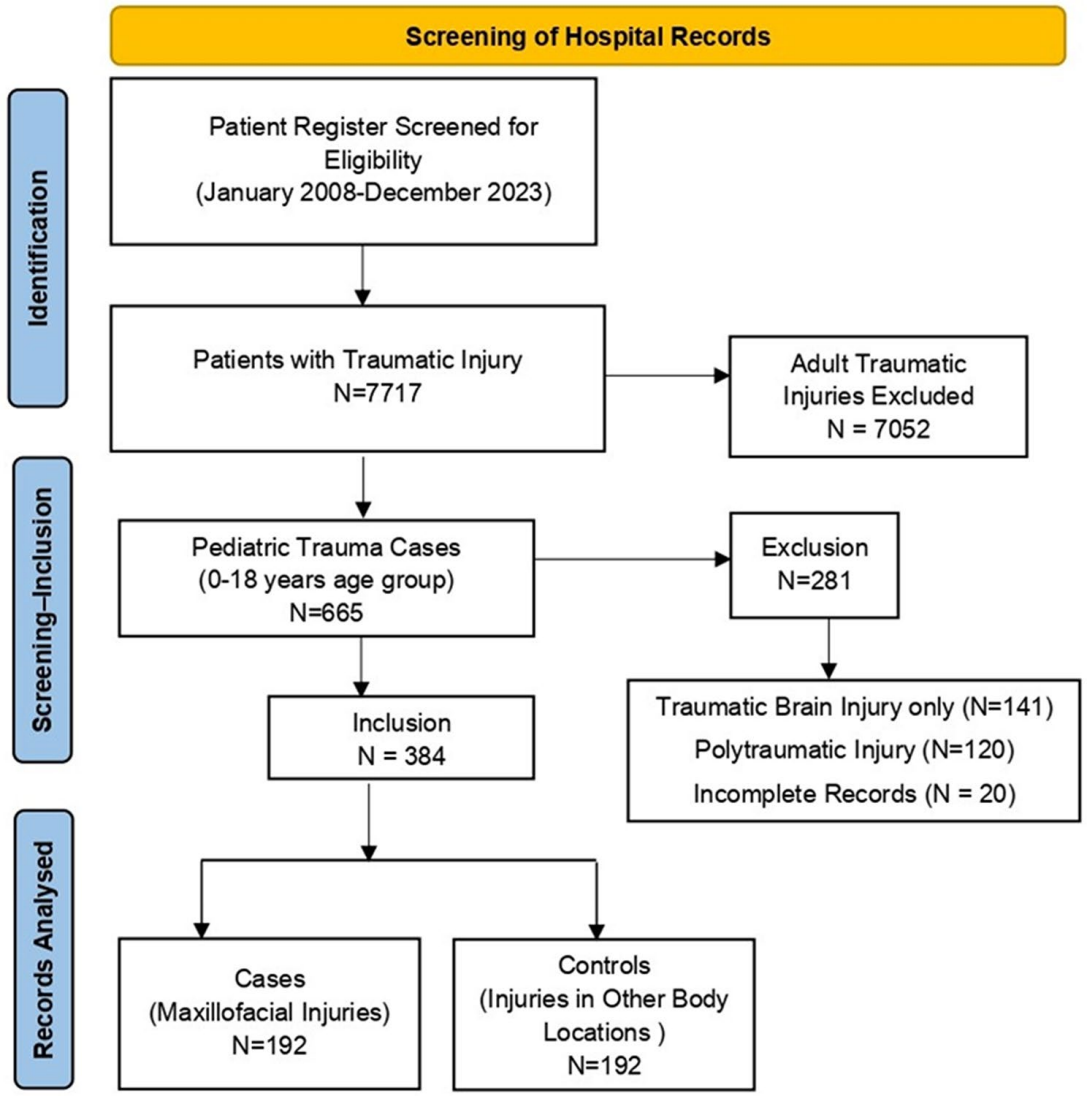
Flow chart depicting the process of inclusion of patient records in the study

The sample included in the study consisted of 192 cases and 192 controls. This sample was included after screening patient records from January 2008 to December 2022 (15 years). The details on the process of inclusion of patient records in the study are provided in the form of a flow chart (Fig. 1). The demographic details of the sample are shown in Table 1. There were no significant differences between the cases and controls in terms of sex, cause of trauma [road traffic accidents (RTA) or not], or medical or drug/alcohol abuse history. Road traffic accidents accounted for most of the traumatic injuries in both cases (78.6%) and controls (85.9%). Among the other causes of trauma, the most common were falls (*n* = 37 and *n* = 21 for cases and controls, respectively), followed by sports injuries (*n* = 3 and *n* = 1 for cases and controls, respectively), work-related injuries (*n* = 1 and *n* = 3 for cases and controls, respectively), and assaults (*n* = 0 and *n* = 2 for cases and controls, respectively). Most of the paediatric trauma cases involved adolescents (12–18 years). A greater number of adolescent individuals were controls than cases, while there were more cases in the 0–5-year-old and 6–11-year-old age groups, and the difference was significant (*p* = 0.007). A greater number of males had traumatic injury in both the case (72.4%) and control (67.4%) groups. The number of patients with a medical history/disability and drug/alcohol abuse was minimal (range 0–4) among both the cases and controls.

**Table 1:** Demographic variables of the sample included in the study

Variable		Case (With maxillofacial injury) (%)	Control (Without maxillofacial injury) (%)	Chi-square value	*p*-value	Case with TBI (%)	Case without TBI (%)	Chi-square value	*p*-value
Age (years)	**0–5**	39 (20.3)	20 (10.4)	9.94	0.007*	21 (20.6)	18 (20.0)	0.74	0.691
	**6–11**	50 (26.0)	42 (21.9)			24 (23.5)	26 (28.9)		
	**12–18**	103 (53.6)	130 (67.7)			57 (55.9)	46 (51.1)		
Sex	**Male**	139(72.4)	129 (67.2)	1.24	0.266	74 (72.5)	65 (72.2)	0.003	0.960
	**Female**	53 (27.6)	63 (32.8)			28 (27.5)	25 (27.8)		
Medical History	**Yes**	7 (3.6)	4 (2.1)	0.84	0.359	6 (5.9)	1 (1.1)	3.10	0.078
	**No**	185 (96.4)	188 (97.9)			96 (94.1)	89 (98.9)		
Disability	**Yes**	0 (0)	1 (0.5)	1.003	0.317	0(0)	0 (0)	…	….
	**No**	192 (100)	191 (99.5)			102 (100)	90 (100)		
Drug/Alcohol Abuse History	**Yes**	1(0.5)	1(0.5)	0.0	1.000	1 (0.5)	0 (0)	0.89	0.346
	**No**	191 (99.5)	191 (99.5)			191 (99.5)	90 (100)		
Cause of Trauma	**Road Traffic Accidents**	151 (78.6)	165 (85.9)	3.50	0.061	78 (86.7)	73 (71.6)	6.49	0.011*
	**Other Causes**	41 (21.4)	27 (14.1)			12 (13.3)	29 (28.4)		
Hospitalization Duration	**≤ 3 Days**	131 (68.2)	141 (73.4)	0.26	0.312	47 (46.1)	84 (93.3)	49.25	< 0.001*
	**≥ 4 Days**	61 (31.8)	51 (26.6)			55(53.9)	6(6.7)		
Total		**192 (100)**	**192 (100)**			**102 (100)**	**90 (100)**		

*p < 0.05: significant

**Fig. 2:**
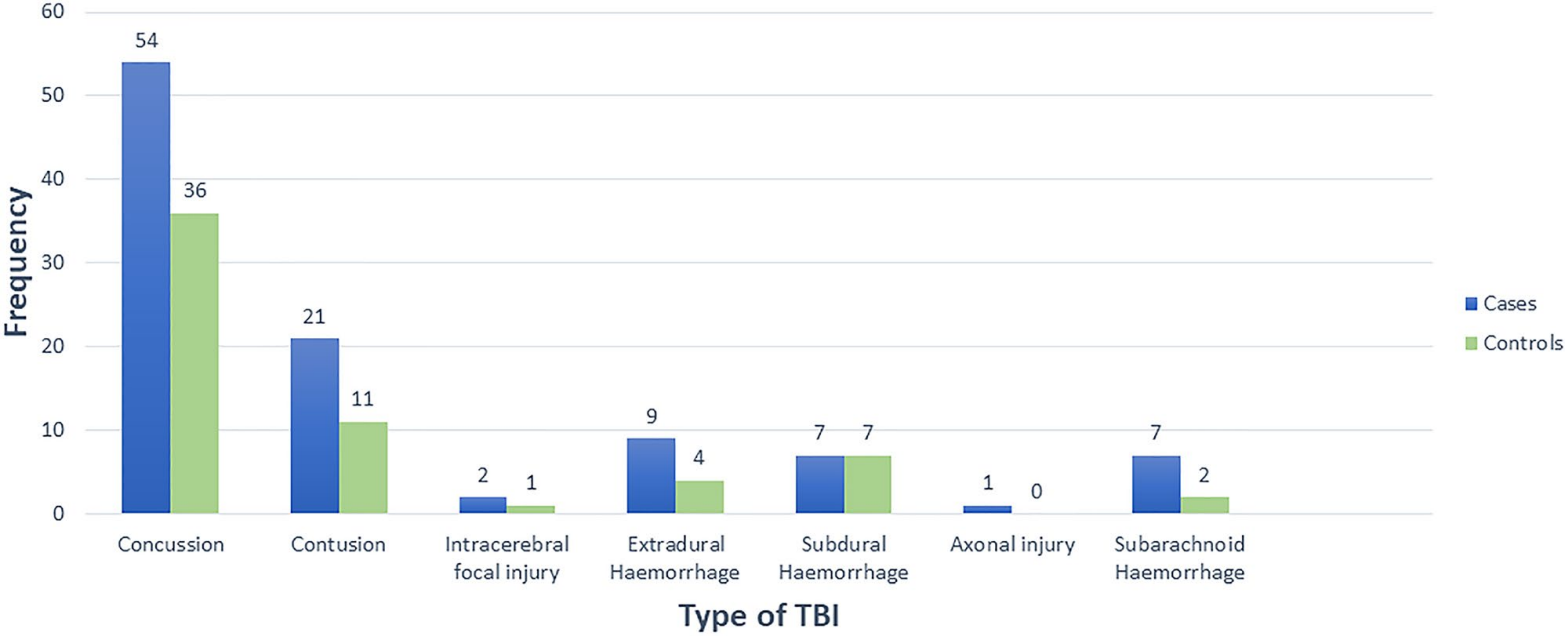
Types of traumatic brain injury among cases and controls

A comparison of cases and controls for TBI occurrence revealed a statistically significant difference, with 53% of cases recording TBI, compared to 32% among controls. The odds ratio indicated that the chance of TBI among the cases was approximately 2.5 times greater than the controls (Table 2). Among the neurological assessment signs, abnormal structural imaging, loss of consciousness, alteration of mental state after trauma and the best available GCS score in the first 24 h were significantly different between the cases and controls (Table 3). The data on severity of TBI based on the neurological assessment revealed that the frequency of moderate TBI was higher in cases than the controls. The frequency of mild and moderate TBI was higher among the cases than the controls and the difference was significant (Table 4). Traumatic brain injury (TBI) included concussion, brain contusion, focal injury, diffuse injury (traumatic axonal injury), neurocranium fracture, and traumatic extradural, subdural, or subarachnoid haemorrhage. Most of the TBIs were concussion injuries in both the case and control groups (Fig. 2), with no significant difference between the cases and controls (χ2 = 4.37; *p* = 0.822).

**Table 2:** Comparison of cases and controls for the association with traumatic brain injury (TBI)

Variable	Case (With maxillofacial injury) (%)	Control (Without maxillofacial injury) (%)	Chi-square value	*p*-value	Odds ratio	Confidence interval (Upper-lower)
TBI Present	102 (53.1)	61 (31.8)	17.92	< 0.001*	2.43	1.60–3.69
TBI Absent	90 (46.9)	131 (68.2)				
Total	192 (100)	192 (100)				

*p < 0.05: significant

**Table 3:** Distribution of TBI signs and symptoms among cases and controls

Variable		Cases (%)	Controls (%)	Chi-square value	*p*-value
Structural Imaging	**Normal**	130 (67.7)	163 (84.9)	15.68	< 0.001*
	**Abnormal**	62 (32.3)	29 (15.1)		
Loss of Consciousness	**0–30 min**	43 (22.4)	25 (13.0)	11.96	0.008*
	**>30 min and < 24 h**	8 (4.2)	2 (1.0)		
	**> 24 h**	0 (0)	2 (1.0)		
	**None**	141 (73.4)	163 (84.9)		
Alteration of mental state immediately after trauma	**Up to 24 h**	5 (2.6)	0 (0)	7.02	0.030*
	**More than 24 h**	0(0)	2 (1)		
	**None**	187 (97.4)	190 (99.0)		
Post-traumatic amnesia	**0–1 day**	9 (4.7)	4 (2.1)	3.02	0.221
	**> 1 day and < 7 days**	1 (0.5)	0 (0)		
	**> 7 days**	0 (0)	0 (0)		
	**None**	182 (94.8)	188 (50.8)		
Glasgow Coma Scale (Best available score in the first 24 h)	**13–15**	179 (93.2)	189 (98.4)	8.6	0.014*
	**9–12**	11 (5.7)	1 (0.5)		
	**≤ 8**	2 (1.1)	2 (1.1)		
Total		**192 (100)**	**192 (100)**		

*p < 0.05: significant

**Table 4:** Severity of TBI among cases and controls

	Cases (%)	Controls (%)	Chi-square value	*p*-value
No TBI	90 (46.9)	131 (68.2)		
Mild TBI	85 (44.3)	57 (29.7)	23.07	< 0.001*
Moderate TBI	15 (7.8)	2 (1.0)		
Severe TBI	2 (1.0)	2 (1.0)		
Total	192(100)	192 (100 )		

*p < 0.05: significant

Further analysis of the factors associated with TBI among the cases revealed a significant association between the cause of trauma, hospitalization duration and TBI among the cases. RTA was significantly greater among the cases with TBI, while other causes of injury (including falls, sports injuries, and assault) were more common among the cases with no TBI. The hospitalization duration was lower among the cases with no TBI (Table 1). Among the symptoms associated with TBI, vomiting, loss of consciousness, headache, convulsions, ear bleeding and nose bleeding were reported significantly more frequently among the cases with TBI (Table 5).

**Table 5:** Symptoms associated with TBI among the cases

Variable		With TBI (%)	Without TBI (%)	Chi-square value	*p*-value
Vomiting	**Yes**	52 (51.0)	4 (4.4)	50.12	< 0.001*
	**No**	50 (49.0)	86 (5.6)		
Loss of Consciousness	**Yes**	40 (39.2)	4 (4.4)	52.72	< 0.001*
	**No**	62 (60.8)	86 (95.6)		
Headache	**Yes**	24 (23.5)	1 (1.1)	21.22	< 0.001*
	**No**	78 (76.5)	89 (98.9)		
Convulsions	**Yes**	8 (7.8)	0 (0)	7.37	0.007*
	**No**	94 (92.2)	90 (100)		
Nosebleed	**Yes**	30 (29.4)	72 (70.6)	28.29	< 0.001*
	**No**	1 (1.1)	89 (98.9)		
Ear Bleed	**Yes**	7 (6.9)	95 (93.1)	3.96	0.047*
	**No**	1 (1.1)	89 (98.9)		
	**Total**	**102 (100)**	**90 (100)**		

*p < 0.05: significant

Among the types of maxillofacial injuries, facial fractures were significantly more common among the cases with TBI. Approximately 40% (*n* = 41) of cases with TBI had facial fracture, while approximately 12% (*n* = 11) of cases without TBI had facial fracture (Table 6). Among the total 52 cases with facial fractures, 63.5% (*n* = 33) had mid-face fractures, 28.8% (*n* = 15) had upper-face fractures, and 7.7% (*n* = 4) had lower-face fractures. Cases with TBI mostly had upper face fractures (*n* = 14, 34.1%), followed by mid-face fractures (*n* = 23, 56.1%). There were only a few lower face fractures (*n* = 4, 9.8%). In comparison, among the cases without TBI, most had mid-face bone fractures (*n* = 10, 90.9%) and the remaining one case had upper face fracture. There was no significant association between facial fracture type and the presence of TBI (χ2 = 4.6; *p* = 0.099).

**Table 6:** Type of maxillofacial injury associated with TBI among the cases

Variable		With TBI (%)	Without TBI (%)	Chi-square value	*p*-value
Facial Fracture	**Yes**	41 (40.2)	11 (12.2)	18.95	< 0.001*
	**No**	61 (59.8)	79 (87.8)		
Dentoalveolar Fracture	**Yes**	2 (2.0)	10 (11.1)	6.83	0.009*
	**No**	100 (98.0)	80 (88.9)		
Soft tissue injuries	**Yes**	78 (76.5)	76 (84.4)	0.21	0.114
	**No**	24 (23.5)	14 (15.6)		
Total		**102 (100)**	**90 (100)**		

*p < 0.05: significant

When the other types of maxillofacial injuries were further analysed for TBI associations, dentoalveolar fracture was significantly associated with cases without TBI while soft tissue injuries were not associated with TBI (Table 6). The mean FISS score for maxillofacial injuries was greater among cases with TBI, and the difference in the FISS score between cases with TBI (mean = 1.31 ± 1.74) and those without TBI (mean = 0.74 ± 0.91), as determined by the nonparametric Mann‒Whitney test, was significant (*p* = 0.019).

Multiple logistic regression analysis with the presence/absence of TBI among the cases as the dependent variable and other independent variables that entered the analysis by the enter method, revealed a significant association of RTA, duration of hospital stays, and dentoalveolar fracture with TBI. Omnibus tests for model coefficients were significant, indicating the validity of the model, and the null hypothesis was rejected. The goodness of fit test (Hosmer‒Lemeshow test) was not significant (χ2 = 7.31, *p* = 0.504), indicating the predictability of the independent variables. The odds ratios indicated that RTA increased the risk of TBI among the cases by 3.5 times, while an odds ratio of 0.04 indicated that dentoalveolar fracture is an indicator of a significantly lower risk for TBI among the cases. Cases with TBI were likely to be hospitalized for greater than four days (Table 7).

**Table 7:** Multiple logistic regression analysis model depicting the odds ratio between the presence and absence of TBI among the cases

Variable		Wald	Odds ratio	*p*-value	Confidence interval (Lower-upper)
Age	**0–6 (ref)**				
	**6–12**	0.28	0.76	0.599	0.28–2.10
	**13–18**	0.11	1.16	0.744	0.48–2.82
Sex	**Female (ref)**				
	**Male**	0.99	0.67	0.321	0.30–1.48
Trauma Cause	**Other Causes (ref)**				
	**Road Traffic Accidents**	7.68	3.51	0.006*	1.44–8.52
Duration of Hospital Stay	**≤ 3 Days (ref)**				
	**≥ 4 Days**	28.62	22.09	< 0.001*	7.11–68.64
Facial Fracture	**No (ref)**				
	**Yes**	0.22	1.38	0.641	0.36–5.32
Dentoalveolar Fracture	**No (ref)**				
	**Yes**	7.86	0.04	0.005*	0.01–0.37
Soft tissue injuries	**No (ref)**				
	**Yes**	0.48	0.60	0.489	0.14–2.59
FISS score		0.02	1.03	0.893	0.71–1.49

*p < 0.05: significant

## Discussion

The present study aimed to evaluate the association between TBI and maxillofacial injuries in a paediatric age group. This was done by comparing the occurrence of TBI in paediatric patients who reported to the hospital with injuries in the maxillofacial region and other locations of the body. The results of this case–control study revealed a positive association between maxillofacial injuries and TBI among paediatric patients. There are multiple published studies on the association between TBI and maxillofacial injuries with varying results. A literature review revealed a greater occurrence of TBI among patients with maxillofacial injuries [9], but there is little or no literature specific to the paediatric age group. In our study, more than 50% of patients with maxillofacial injuries were diagnosed with TBI. The greater risk of TBI among patients with maxillofacial injuries seems to be due to the close anatomic proximity of the maxillofacial region to the cranium [15, 16]. The results of our study are similar to those of Rajandram et al., who reported that the risk of TBI increased by 1.5 times among patients with facial fractures; however, their study sample included a wide age range of 2–86 years, and only approximately 35% of the patients were in the paediatric age group [8]. The risk of occurrence of TBI in our study was 2.5 times greater among paediatric patients with maxillofacial injuries than among those with injuries involving other parts of the body. Maxillofacial injuries, along with TBI, may cause serious aesthetic and functional deficits in a growing child. The presence of injuries in the orofacial region draws immediate attention from the patient, primary care givers and treating surgeons/physicians. However, the diagnosis of TBI is challenging in the paediatric population due to diverse clinical presentations in this age group, potentially leading to missed diagnoses of TBI [13]. As there is some evidence regarding the association between maxillofacial injuries and TBI; in cases of maxillofacial injuries in paediatric age group, TBI needs to be thoroughly investigated to avoid missed or late diagnosis.

Among the 192 children with maxillofacial injuries, more than half were adolescents. Risk-taking behaviour seems to increase as a child reaches adolescence [17]. An earlier study reported that children in the 11- to 15-year age group were more likely to experience paediatric trauma than were those in other age groups. Reduced parental control and increased urge to be independent may lead to risk-taking behaviour among older children [17]. Our study data also revealed that most paediatric trauma patients were between 12 and 18 years of age. However, in the case group, the occurrence of trauma was greater in the 0-5- and 6-11-year age groups. Trauma predilection for craniofacial injuries in younger age groups is generally attributed to a greater head-to-body weight ratio and weaker neck musculature [18]. However, age did not significantly influence the occurrence of TBI in the case group.

Consistent with the findings of other studies, males were more prone to traumatic injuries than females in both the case and control groups, though the difference was not statistically significant [9, 17]. This is probably attributed to more risk-taking attitudes and outdoor activities among boys. The cultural role of males may also expose them to potentially risky environments. This social factor needs to be addressed to formulate strategies to prevent injuries among boys. In our study, only one patient in each group had a history of drug and alcohol use. The screening rates for alcohol and drug abuse remain low for paediatric trauma patients and guidelines for drug and alcohol screening, including blood and urine testing should be established for trauma patients in hospitals so that underestimations can be avoided [19]. This may help in strategizing the prevention of trauma in this age group.

The association of RTA with maxillofacial injuries with TBI has been observed in studies that included a wide age range in the sample, both children and adults [8, 20]. Like other studies, we saw RTAs as a major cause of trauma in the paediatric age group. A total of 78.6% of the children with maxillofacial injuries (cases) and 85.9% of the children in the control group had RTA as the etiologic factor. When the factors influencing the occurrence of TBI among patients with maxillofacial injuries were further analysed by regression analysis, RTA was identified as a risk factor. RTA is a known risk factor for both TBI and maxillofacial injuries [21–23].

The presence of TBI with maxillofacial injuries indicated prolonged hospitalization for the paediatric patients in our study. In our study, we observed that most of the patients with maxillofacial trauma without TBI (93.3%) had a hospital stay of less than 3 days. Whereas only 46.1% of the maxillofacial trauma patients with TBI stayed in the hospital for less than 3 days, more than 50% of the patients had a longer hospital stay, with more than 15% of this group of paediatric patients staying in the hospital for more than 8 days. Multicentric studies have shown that the mean duration of hospitalization for patients with TBI for acute care is 7 to 13 days. In general, the length of hospitalization increases with increasing severity of TBI [24–26].Although the age, sex, and socioeconomic status of the participants could have influenced the duration of hospitalization, the presence of TBI along with maxillofacial injuries indicates a greater need for surgical intervention and increased risk of infection [27] Patients with TBI are hospitalized for observation, stabilization, and neurosurgical interventions [28]. The experience of hospitalization has psychological, emotional, and physical impacts, as it can be one of the most distressful events in one’s lifetime. Hospitalized children experience disruption of routines and stress. Since children have limited coping abilities and emotional resources, extended hospital stay can have major short- and long-term impacts [29].

A definite association between facial fractures and TBI has long been debated. Although the occurrence of TBI was greater among patients with facial fractures and significant in the binary analysis, this difference was not significant when other factors were accounted for in the regression analysis. However, it was interesting to note that the risk of TBI was significantly lower among patients with dentoalveolar injuries. In our study, the odds ratio for the association between dentoalveolar fractures and TBI was 0.04, suggesting that dentoalveolar injury is a protective factor and that patients with only dentoalveolar injury may not be at risk for TBI. Among the 12 patients with dentoalveolar injury in our study, only 2 had TBI. Both patients had severe mid-face fractures.

Earlier studies involving adult individuals revealed a significant association between facial fractures and TBI [8, 20], while Keenan et al. [30] reported no such association. It is also debated whether facial fractures occurring due to blunt impact, can have a cushioning effect on the brain, depending on the site of the facial fracture. In such cases maxillofacial fractures dissipate forces, leading to less severe TBI [8]. The same was reflected in our study data, as despite the higher frequency of TBI among the cases, in terms of severity, mild and moderate TBI were higher among cases than the controls.

While upper-face and mid-face fractures have been associated with TBI, they are less likely to occur with mandibular fractures [19, 31]. A prospective study revealed a clear association between fractures present in the upper and middle thirds of the face and TBI. A high incidence of TBI has been associated with frontal bone and zygomatic complex fractures. Additionally, complex maxillofacial injuries with multiple bone fractures are at increased risk for TBI [28]. In a case series of 100 patients comprising both children and adults, all facial fracture cases were associated with TBI, and most of them were in the mid-face region [32]. The anatomic proximity of the traumatic injury seems to be a risk factor for TBI. In our study, midface and upper fractures were more common among patients with TBI than among patients without TBI, although the difference was not significant. Among the four patients with mandibular fracture with TBI, three had condylar fractures (proximity to the skull base), and one patient had associated zygomatic complex and nasal bone fractures. Subgroup analysis of the patients with facial fractures in our study revealed that most of the injuries involved the mid-face region among both children with and without TBI; hence, an association between facial fracture type and TBI could not be established. The lack of association could be due to the smaller proportion of the sample with facial fractures. As only 52 patients were diagnosed with facial fractures, the small subset of the study sample was a limitation of the study data. However, our study revealed a significant association between an increase in the severity of facial trauma, as assessed by the FISS, and the presence of TBI. Hence, complex maxillofacial injuries, involving the upper and midface, may be considered a risk factor for TBI.

Among the cases, soft tissue injuries were not associated with TBI. Earlier studies have yielded conflicting results on the association of soft tissue injuries with TBI. A cohort study revealed no association of soft tissue injuries with or without facial fractures with TBI [12], while a case control study found that 11.2% of patients with both facial bone fractures and/or soft tissue injuries had concomitant head injuries and 13% of patients who had soft tissue injuries alone also had TBI [16]. In another study, all types of maxillofacial injuries had association with mild TBI, which included 18.6% of soft tissue injuries alone [8]. The difference in results may be because the sample in our study involved pediatric age group, while others involved adults, with wide age range. The association of soft tissue injuries with TBI was not established by our study data.

In our study, there was a significant association of signs of TBI such as loss of consciousness, alteration of mental state immediately after trauma, and GCS score, with maxillofacial injury. An earlier study reported that 38.7% of maxillofacial trauma patients reported loss of consciousness, which ranged from less than 30 min to more than 6 h [33]. Among the 192 children who experienced maxillofacial trauma in our study, more than 22% reported loss of consciousness for less than 30 min. Only 4.2% reported a loss of consciousness lasting more than 30 min. Most patients with maxillofacial trauma (93.2%) had GCS scores of 13–15, and the proportion was similar to that of patients who presented with injuries outside of the maxillofacial region (98.4%). Notably, a GCS score between 9 and 12 was observed in 11 (5.7%) maxillofacial trauma patients compared to only 1 (0.5%) in the control group.

Clinical diagnosis of TBI is based mostly on the patient’s history and clinical signs [28]. We noted that among the cases with TBI, loss of consciousness, vomiting, headache, convulsions, and nasal and ear bleeding were significantly more common and thus, a positive history of these symptoms should alert the clinicians to the possibility of TBI. A prospective study reported a 67% incidence of head injury associated with maxillofacial trauma. Furthermore, among the patterns of head injury, concussion was most strongly associated with maxillofacial trauma [34]. Similar findings were observed in our study. We observed that more than half of the patients with TBI in both the case and control groups had concussion injuries. More than 80% of the patients in the case group had mild TBI. Facial fractures, particularly those with multiple injuries, have been associated with mild TBI [35]. The diagnosis of mild TBI is important, particularly if it is associated with maxillofacial injury, as the risk for neuropsychological and cognitive impairment is greater [5].

Thus, based on our study results during the evaluation of paediatric patients with maxillofacial injuries, the presence of facial fractures should alert clinicians to the possibility of TBI, particularly mild TBI such as concussion injuries. It is important to obtain a history of symptoms related to TBI in these patients. In contrast, mild maxillofacial injuries such as dentoalveolar fractures indicate a lower likelihood of TBI. However, we recommend that all paediatric patients with maxillofacial injuries undergo further investigation via CT scan and clinical observation for TBI, considering the greater odds of TBI observed in our study.

Prevention of TBI injury strategies for children should focus on male children, particularly those less than 12-years of age who may be at greater risk for maxillofacial injuries and hence TBI, screening adolescents for drug and alcohol history and prevention of RTA. Such measures will prevent prolonged hospitalization associated with TBI that can affect the psychological well-being of children.

The current study was conducted in a single tertiary care hospital and hence may not represent all the paediatric trauma cases reported in the region. The study design is retrospective and is dependent on the accuracy of patient records for data on patient history, clinical signs, and diagnosis. Any errors in the original examination or documentation influenced the study outcomes. We could not find follow-up data from the patients’ records, limiting the availability of data on possible late TBI diagnoses. However, we analysed paediatric trauma cases over a 15-year period with a well-defined sample size and conducted this study with a case‒control design, taking into consideration the lower occurrence of trauma in the paediatric age group in earlier studies involving wide age ranges [8, 32]. The case–control design allows for obtaining odds ratios that quantify the risk of TBI. We did not match the age or sex of the patients, which are common variables used for matching, as the available literature indicated that age and sex are strong risk factors for TBI [8, 16, 17, 36]. The confounding effect of the age and sex factors was adjusted for during the logistic regression analysis. The results of our study provide only an association of the possible risk factors for the occurrence of TBI among children with maxillofacial injuries, due to the inherent temporal bias of the case-control design. Further prospective studies focusing on accurate and standardized multicentric data recording systems will provide a more definitive conclusion on whether all paediatric patients with maxillofacial injuries should be screened for TBI. Conversely, future studies can also investigate if paediatric patients with TBI need to be screened for maxillofacial injuries.

## Conclusion

Within the limitations of the study design, the following conclusions may be drawn:

1. Paediatric patients with maxillofacial injuries need to be screened for TBI, as the risk of occurrence of TBI is 2.5 times greater among paediatric patients with maxillofacial injuries than among those with injuries involving other parts of the body.
2. RTA and prolonged hospitalization are factors associated with TBI among paediatric patients with maxillofacial injuries. The presence of only dentoalveolar injuries decreases the likelihood of a TBI.

## Data Availability

The datasets used and/or analysed during the current study are available from the corresponding author on reasonable request.

## Abbreviations

TBI: Traumatic Brain Injury
RTA: Road Traffic Accident
GCS: Glasgow Coma Scale
CT scan: Computed Tomography Scan
FISS: Facial Injury Severity Scale
SPSS: Statistical Package for Social Sciences
